# Moving4notfrail^®^: A Rehabilitation Nursing Programme for Older Adults with Frailty

**DOI:** 10.3390/nursrep15120419

**Published:** 2025-11-27

**Authors:** Ana Isilda Torres Martins Santos, Ana da Conceição Alves Faria, Carla Gomes da Rocha, Abel Fernandes, Mariana Filipa Mendes Gonçalves, Joana Isabel Alves Quintas, Maria Narcisa da Costa Gonçalves, Olga Maria Pimenta Lopes Ribeiro

**Affiliations:** 1 Department of Nursing, Tâmega and Sousa Local Health Unit, 4564-007 Guilhufe, Portugal; anaisildasantos@gmail.com; 2 Department of Nursing, Santa Maria Health School, 4049-024 Porto, Portugal; ana.faria@santamariasaude.pt; 3 Department of Nursing, School of Health Sciences, HES-SO Valais-Wallis, 1950 Sion, Switzerland; carla.gomesdarocha@hevs.ch; 4 Department of Nursing, Gaia and Espinho Local Health Unit, 4434-502 Vila Nova de Gaia, Portugal; 5 Department of Nursing, Lusíadas Lisbon Hospital, 1500-458 Lisboa, Portugal; 6 Department of Nursing, Nursing School of Porto, 4200-072 Porto, Portugal; 7 RISE-Health, 4200-319 Porto, Portugal

**Keywords:** rehabilitation nursing, frail older adults, physical frailty, muscle strength, gait, Delphi technique, hospitalization

## Abstract

**Background/Objectives**: Population ageing and the need for hospitalisation due to acute or chronic illness have contributed to increased physical frailty among older adults, with implications for their quality of life and healthcare. This study aims to describe the development and validation process of a rehabilitation nursing programme for hospitalised older adults experiencing physical frailty. **Methods**: The e-Delphi study was conducted between September 2024 and May 2025, comprising three phases: (1) development of the rehabilitation nursing programme for frail older adults admitted to hospital; (2) validation of the programme content using a modified e-Delphi technique; and (3) development of the final programme prototype. **Results**: A panel of 18 experts participated. After a pair of rounds, every single program component achieved a Content Validity Index CVI over or equal to 0.90, and expert agreement was 100% related to the possibility of preventing frailty. The resulting prototype, Moving4notfrail^®^, includes a progression of muscle-joint exercises in five positions (lying down, sitting in bed with feet on the floor, sitting in a chair, standing and walking). It integrates strategies such as dual tasks, sensory and visual stimuli, and verbal and tactile guidance to enhance participant engagement and adherence to the programme. **Conclusions**: The experts’ contributions validated the rehabilitation nursing programme for frail hospitalised older adults. The final prototype systematises key exercises aimed at preventing the progression of physical frailty and may also serve as a valuable tool in preventing its onset.

## 1. Introduction

As in other countries, population ageing has been one of the main demographic trends in Portugal over the last decade [1]. This has not been accompanied by an improvement in the indicator used to assess the population’s well-being and quality of life [2].

According to the World Health Organization (WHO) [3], by 2050, one in six people in the world will be aged 60 years or over, and the number of adults over 80 will triple, reaching approximately 426 million. This global demographic transition represents a major public health challenge, given the higher prevalence of chronic diseases, multimorbidity, disability, and frailty associated with the ageing process.

Ageing is a natural part of the life course, but increased longevity poses challenges to healthcare planning and delivery due to the higher prevalence of chronic diseases and age-related clinical conditions [3]. Over the past decade, this has become a significant issue in the field of nursing [4]. The World Health Organization (WHO) itself, as part of the United Nations Decade of Healthy Ageing 2020–2030, has been making efforts to ensure that the ageing of the world’s population is perceived as an opportunity rather than a challenge [5].

Throughout the natural ageing process, some individuals remain relatively healthy and resilient, while others become frail and, consequently, more vulnerable to internal and external stressors, placing them at greater risk of adverse outcomes such as falls, fractures, hospitalisations, postoperative complications, institutionalisation, disability, and even death [6,7]. Ageing does not inevitably lead to frailty, but with advancing age, this condition tends to become more prevalent [8,9].

Frailty is a dynamic, multifactorial state affecting individuals who experience losses in one or more domains of human functioning—physical, psychological, or social [6]. The WHO conceptualises this condition as a clinically recognisable state of increased vulnerability to stressors in older adults, resulting from a diminished ability to maintain homeostasis due to a decline in physiological reserves [9]. Frailty is thus an insidious and silent continuum, preceded by states of robustness and pre-frailty.

Given the multidimensional nature of frailty, its management poses a challenge for professionals, as it requires a holistic, personalised, and integrated approach, as well as the definition of the most appropriate interventions and the optimal timing for their implementation [7,10]. Evidence suggests that appropriate interventions within a rehabilitation plan can prevent, or at least partially reverse, frailty [10].

Identifying frailty in older adults is crucial to the success of the rehabilitation plan [10]. A considerable number of tools are currently available to assess frailty, and their selection should primarily be guided by the underlying conceptual model and the specific context of use [9]. In the physical domain, the Fried frailty phenotype is one of the main assessment tools, indicating frailty when three or more phenotypic criteria are present: unintentional weight loss, weakness, reduced walking speed, low physical activity, and decreased muscle strength [11].

Specialist Nurses in Rehabilitation Nursing (SNRN) frequently interact with frail older adults or those in a pre-frailty stage, regardless of the level of care provided. At the hospital level, regardless of the underlying disease aetiology, hospitalisation in older adults often leads to functional decline, negatively affecting their ability to perform self-care activities [12]. The development or exacerbation of frailty in hospital settings has been one of the primary concerns of SNRN [13]. These professionals play a key role in the early identification of older adults’ health needs and in the design and implementation of care plans and programmes aimed at maximising functional capacity, preventing age-related complications, and promoting social inclusion and participation [14]. Despite the existence of regulations, the systematisation of SNRN interventions in the prevention of frailty among hospitalised older adults remains limited and focused on specific cases [15]. Considering the above and the need for greater investment in this area, this study, as part of a broader project, aims to describe the development and validation process of a rehabilitation nursing programme for hospitalised older adults experiencing physical frailty.

## 2. Materials and Methods

This research followed an e-Delphi design [16], focusing on the development and validation of a rehabilitation nursing programme for frail older adults admitted to hospital. The process was conducted in three phases: (1) development of the rehabilitation nursing programme tailored to hospitalised frail older adults; (2) content validation of the programme through a modified e-Delphi technique; (3) development of the final programme prototype.

### 2.1. Study Design

In Phase I, a rehabilitation nursing programme for frail older adults hospitalised in an acute care setting was developed. In this initial phase, the content to be included was informed by a scoping review (registered in the Open Science Framework: https://doi.org/10.17605/OSF.IO/ADPEJ), previous studies with the participation of older adults [17,18], regulatory frameworks governing the professional practice of SNRN in Portugal [14,19,20], as well as the professional expertise of the authors, who are SNRN.

The developed rehabilitation nursing programme integrated personalised interventions targeting muscle strength and walking—domains particularly sensitive to the expertise of SNRN and identified, according to the phenotypic frailty model [11], as key contributors to frailty. Upon completion of this phase, it became evident that the programme required expert validation to ensure that its components were aligned with the realities of rehabilitation nursing care for frail older adults in hospital settings.

Thus, in Phase II, a modified e-Delphi study was conducted to validate the programme content. Expert consensus was achieved after two rounds. To ensure methodological rigour, the study was carried out in accordance with the Conducting and Reporting of Delphi Studies (CREDES) recommendations [21].

In Phase III, following the validation of the programme content, the final prototype was developed. As one of the main objectives was to promote the implementation of this type of programme in a hospital setting and facilitate its dissemination among professionals and older adults, all exercises were documented through photographs.

### 2.2. Participants

In Phase II, an intentional non-probabilistic sampling technique was used to select the experts. In this study, “experts” were defined as professionals with extensive knowledge and experience in rehabilitation nursing care for older adults. Potential participants were identified through professional networks and recommendations from academic peers. The inclusion criteria were:# (a) being a nurse specialising in rehabilitation nursing; (b) having experience/knowledge of or active involvement in research projects related to rehabilitation nursing care for older adults; and (c) being available and willing to complete all the necessary rounds of the e-Delphi process. A total of 20 experts were selected. Participants were contacted via email, which included a formal invitation letter and a link to access the study. Upon accessing the study, they received information regarding the study’s objectives, rationale, potential benefits, and informed consent, which they had to accept before starting the questionnaire. Anonymity and confidentiality were ensured, and participants were informed of their right to withdraw from the study at any time without any repercussions.

### 2.3. Data Collection and Analysis Procedures

In Phase II, when conducting the modified e-Delphi, it was assumed that consensus reached by a group is more reliable than individual opinions. The content of the rehabilitation nursing programme for frail older adults admitted to hospital was evaluated by the experts using a four-point Likert scale: (1) strongly disagree, (2) disagree, (3) agree, and (4) strongly agree. The expert validation data were organised, and the Content Validity Index (CVI) was calculated by dividing the number of “agree” and “strongly agree” responses (3 and 4) by the total number of responses [15]. Items with a CVI below 0.90 were revised or reformulated, as a CVI of at least 0.90 is recommended [16].

#### 2.3.1. First Round

The questionnaire was developed using Microsoft Forms and comprised two sections: the first collected sociodemographic and professional data, while the second presented the content of the rehabilitation nursing programme for older adults with physical frailty admitted to hospital. For items rated as “strongly disagree” or “partially disagree”, experts were encouraged to suggest improvements. At the end of the questionnaire, all experts had the opportunity to provide additional suggestions. It is worth noting that the questionnaire was pre-tested with five nurses who met the inclusion criteria but were not part of the study sample. This pre-test allowed for the assessment of the comprehensibility of the questions. Based on the CVI calculations and the experts’ suggestions, each item of the programme content was analysed individually in order to make the necessary revisions.

#### 2.3.2. Second Round

After incorporating the feedback from the first round, a revised version of the programme content was sent to the experts in the second round. All components of the programme were validated in the same way as in the first round, allowing the assessment of stability. As in the first round, at the end of the questionnaire, all experts were invited to provide suggestions. The scores and comments were collected and analysed to determine the content validation provided by the experts. Descriptive statistics were used to analyse the participants’ sociodemographic and professional characteristics and calculate the CVI.

### 2.4. Ethical Considerations

The development and validation of the rehabilitation nursing programme for older adults with physical frailty admitted to hospital is part of a broader research project, which received ethical approval from the Ethics Committee (reference number 024/2020). It is important to note that all participants provided informed consent, and the data collected were treated confidentially.

## 3. Results

The results are presented according to the three phases: (1) development of the rehabilitation nursing programme for older adults with physical frailty admitted to hospital; (2) modified e-Delphi study to validate the programme content; (3) development of the final prototype of the programme.

### 3.1. Phase I—Development of the Rehabilitation Nursing Programme for Older Adults with Physical Frailty Admitted to Hospital

In the first phase of the study, a rehabilitation nursing programme was developed for older adults with physical frailty admitted to hospital. This programme was the result of integrating the best available scientific evidence, regulatory guidelines, and specialised clinical expertise, enabling the design of a structured, person-centred intervention tailored to the specific needs of older adults.

The programme adopts an individualised, periodized, and progressive approach. Each session for older adults with physical frailty admitted to hospital consists of five steps: (a) muscle–joint exercises performed while lying down; (b) muscle–joint exercises performed while sitting on the bed with feet supported on the floor; (c) muscle–joint exercises performed while sitting in a chair; (d) muscle–joint exercises performed while standing; (e) muscle–joint exercises performed while walking.

The proposed frequency of implementation is three to four times per week, with 8 to 12 repetitions per exercise. Progression, through gradual increases in volume, intensity, and complexity, depends on individual tolerance and should be tailored to each person’s specific objectives. The implementation and monitoring of the effects of the program will be the responsibility of the Specialist Nurse in Rehabilitation Nursing.

The developed programme was named “Moving for not frail: rehabilitation nursing programme”, with the acronym “Moving4notfrail”.

### 3.2. Phase II—Content Validation of the “Moving for Not Frail: Rehabilitation Nursing Programme”

In the first round of the modified e-Delphi, 20 experts were contacted and invited to participate, of whom 18 accepted and completed the questionnaire, resulting in a 90% response rate. In the second round, all experts responded within the defined timeframe. Table 1 presents the sociodemographic and professional characteristics of the experts who participated in both rounds of the modified e-Delphi.

In the first round, all experts agreed with the designation “Moving for not frail: rehabilitation nursing programme” and with the acronym “Moving4notfrail”.

Regarding the question “the programme meets the needs of frail older adults in hospital settings,” a 94.4% level of agreement was obtained.

With respect to the programme content, the CVI was calculated. Based on the content validation, components that individually obtained a CVI below 0.90 were reviewed and adjusted. Accordingly, and following the improvement suggestions provided by the experts, two components were refined: the resources used in the programme and the variety of exercises. After incorporating these suggestions, the second round was conducted. The adjustments made to these two components resulted in an improved CVI, which exceeded 0.90 for all components (Table 2).

The implementation of the programme’s exercises in five steps, with sessions delivered three to four times per week, each lasting 30 to 45 min, achieved 100% agreement among the experts. Similarly, progression through gradual increases in exercise volume, intensity, and complexity, tailored to individual tolerance and specific objectives, also received 100% agreement.

Following the suggestions for improving the resources used and the variety of exercises, the programme was refined, compared with its initial version, to include exercises employing different strategies, such as dual-task activities, sensory/visual cues, and verbal/tactile prompts, to promote engagement, motivation, and adherence among older adults.

Based on the experts’ suggestions, the exercise “dragging the cones with the foot along a straight line towards parallel cones while following the Timed Up and Go circuit” was removed from the programme, as it presented a level of complexity beyond what is typically feasible for hospitalised older adults.

In both rounds, the statement “the Moving for not frail: rehabilitation nursing programme has the potential to prevent frailty in older adults resulting from hospitalisation” received 100% agreement.

### 3.3. Development of the Final Prototype of the “Moving for Not Frail: Rehabilitation Nursing Programme”

**Figure 1:**
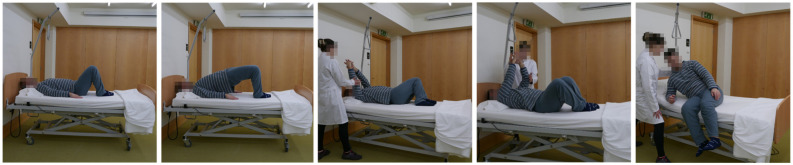
Moving for not frail: rehabilitation nursing programme^®^—Muscle–joint exercises performed while lying down. Note: The two participants shown above are the authors of this article.

**Figure 2:**
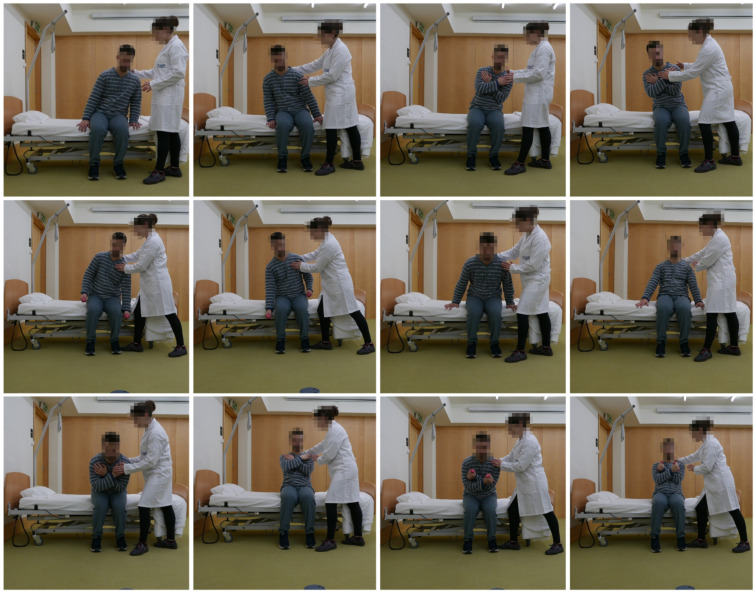
Moving for not frail: rehabilitation nursing programme^®^—Muscle–joint exercises performed while sitting on the bed with feet supported on the floor. Note: The two participants shown above are the authors of this article.

**Figure 3:**
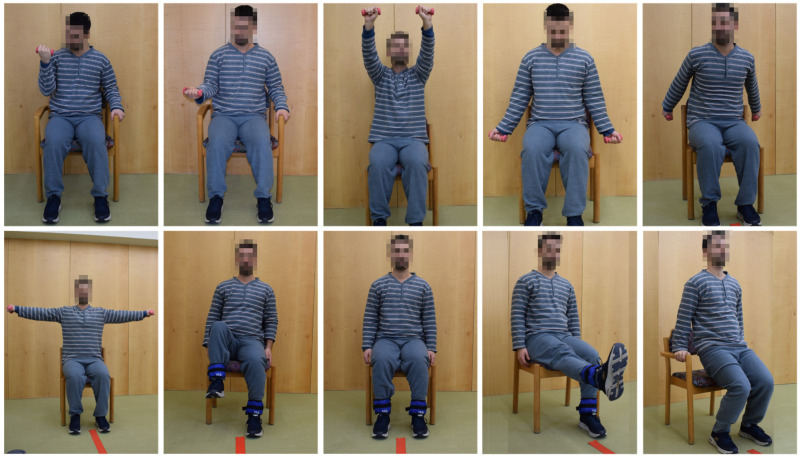
Moving for not frail: rehabilitation nursing programme^®^—Muscle–joint exercises performed while sitting in a chair. Note: The two participants shown above are the authors of this article.

**Figure 4:**
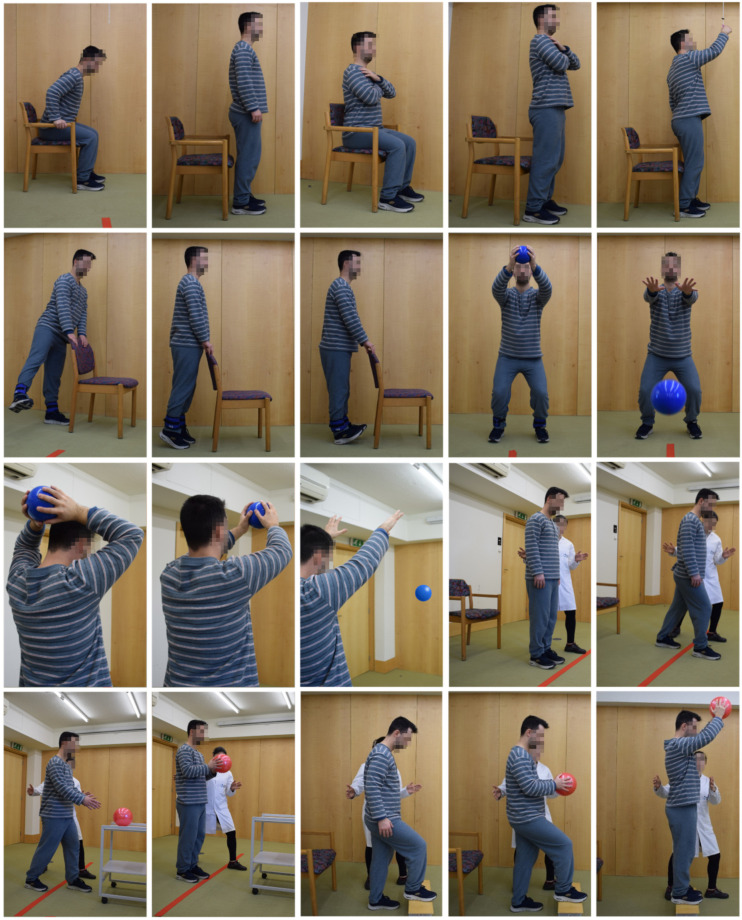
Moving for not frail: rehabilitation nursing programme^®^—Muscle–joint exercises performed while standing. Note: The two participants shown above are the authors of this article.

**Figure 5:**
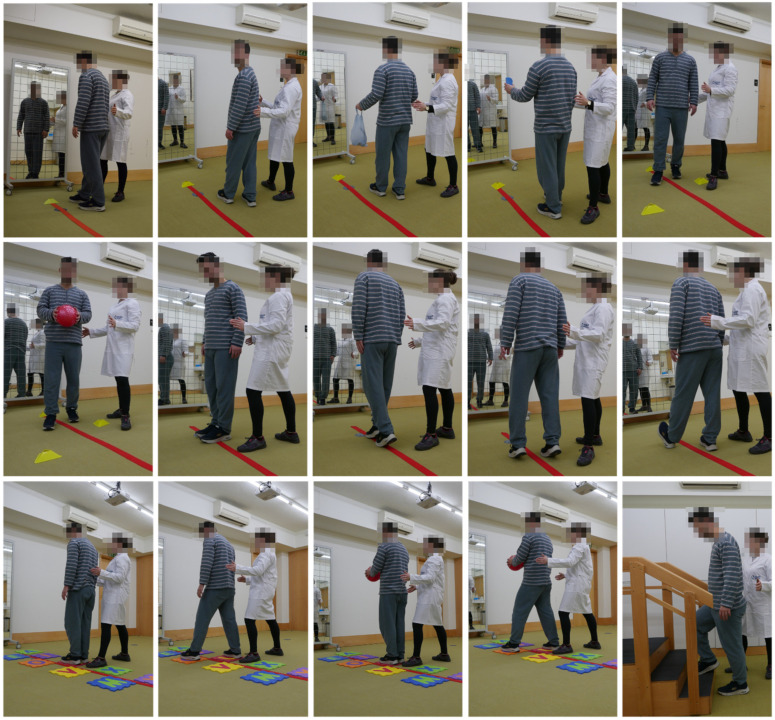
Moving for not frail: rehabilitation nursing programme^®^—Muscle–joint exercises performed while walking. Note: The two participants shown above are the authors of this article.

In the final prototype of the “Moving for not frail: rehabilitation nursing programme” (acronym: “Moving4notfrail”), presented in Figure 1, Figure 2, Figure 3, Figure 4 and Figure 5, the following were outlined: (a) muscle–joint exercises performed while lying down; (b) muscle–joint exercises performed while sitting on the bed with feet supported on the floor; (c) muscle–joint exercises performed while sitting in a chair; (d) muscle–joint exercises performed while standing; (e) muscle–joint exercises performed while walking.

The “Moving for not frail: rehabilitation nursing programme” (Moving4notfrail^®^) is registered with the Portuguese Inspectorate-General for Cultural Activities (IGAC) under work registration number 1443/2024. It is also registered as a National Trademark (No. 726120) with the Portuguese Institute of Industrial Property, under Class 41 of the Nice Classification.

Regarding the programme components, Step I of Moving4notfrail^®^, which focuses on muscle–joint exercises performed while lying down, includes the bridge, pelvic dissociation, and trunk elevation with weight bearing on the elbow (Figure 1).

Steps II of Moving4notfrail^®^, which focuses on muscle–joint exercises performed while sitting on the bed with feet supported on the floor, includes lateral trunk inclinations to the left and right with upper-limb support, without support, and with a 0.5 kg load as well as anterior and posterior trunk flexion with upper-limb support, without support, and with a 0.5 kg load (using dumbbells) (Figure 2).

Step III of Moving4notfrail^®^, which focuses on muscle–joint exercises performed while sitting in a chair, includes elbow flexion and extension with a 0.5 kg load (using dumbbells); shoulder flexion, extension, hyperextension, abduction, and adduction with a 0.5 kg load (using dumbbells); and hip flexion and extension, as well as knee flexion and extension, with a 0.5 kg load (using 0.5 kg ankle weights), concluding with push-ups (Figure 3).

Step IV of Moving4notfrail^®^, which focuses on muscle–joint exercises performed while standing, includes sit-to-stand exercises with and without support; sit-to-stand exercises with a dual motor task: applying clips to a support placed within functional reach; hip abduction and adduction with hands supported on the chair; plantar flexion and dorsiflexion of the ankle joint with hands supported on the chair; throwing a medicine ball to the floor while flexing the knees; throwing a medicine ball overhead against the wall; stepping over a line marked on the floor in front of the feet by advancing one lower limb, shifting weight and bearing support on that limb, then returning to the starting position and repeating with the other limb; the same exercise with an added dual task: during the weight-bearing phase, grasping a ball placed on the table in front of the participant with both hands; stepping onto a step placed in front of the feet by advancing one lower limb, shifting weight and bearing support on that limb; the same exercise with an added dual motor task: holding a ball with both hands and performing shoulder flexion while stepping up (Figure 4).

Step V of Moving4notfrail^®^, which focuses on muscle–joint exercises performed while walking, includes a 3-metre circuit (Timed Up and Go) for gait instruction and training, incorporating dual motor tasks such as walking with cervical rotation (“window shopping”), carrying weighted bags (“carrying groceries”), or transporting a glass of water without spilling it, and dual cognitive–motor tasks such as maintaining verbal communication during the walk or counting steps; navigating obstacles along the 3-metre circuit (Timed Up and Go), with dual motor tasks: as moving the ball from one hand to the other, lifting the ball overhead, or passing the ball around the waist; lateral walking along a marked line and progressing to tandem walking, tiptoe walking, and heel walking using the Timed Up and Go circuit; walking along marked spots on the Timed Up and Go circuit, with dual motor tasks such as holding a ball with both hands and dual cognitive tasks such as identifying the colour of the marking to be stepped on; ascending and descending stairs, with dual cognitive–motor tasks such as maintaining verbal communication or counting the steps (Figure 5).

It is important to note that throughout all steps of the programme, for exercises involving the use of dumbbells and ankle weights as a strategy to increase resistance, load progression should respect individual tolerance and each person’s specific objectives. A gradual increase in weight is recommended, starting at 0.5 kg and progressing to 1 kg, 2 kg, and, eventually, 3 kg.

## 4. Discussion

This study aimed primarily to develop and validate a rehabilitation nursing programme for older adults with physical frailty admitted to hospital. The Moving4notfrail^®^ programme is innovative, distinguished by its progressive structure, personalised approach, and foundation in up-to-date scientific evidence. Beyond the creation of an exercise programme, its main value lies in the expert systematisation and validation of a structured tool, addressing a significant practical gap in the clinical field of rehabilitation nursing. The rigour of the validation process, conducted with a panel of rehabilitation nursing experts, reinforces the robustness and clinical applicability of the programme.

The content validation technique used, the modified e-Delphi, proved appropriate for the purposes of the study. The use of the online format facilitated the recruitment and participation of the experts, enabling the collection of relevant contributions within a short timeframe. Despite the density of the content, a high adherence rate and absence of dropouts were observed, reflecting the experts’ interest in and commitment to the topic. The stability of the responses, combined with the relevance of the suggestions provided, was key to refining and validating the programme components, all of which achieved a CVI of ≥0.90.

The development of Moving4notfrail^®^ responds to the phenomenon of population ageing, particularly evident in Portugal, which has resulted in a growing demand for health care that is specific and adapted to the needs of the older population [2]. Although longevity is increasing, it is not always accompanied by quality of life, with frailty remaining a highly prevalent condition [22,23,24]. It is therefore imperative to develop interventions that not only slow the progression of frailty but also enhance its potential reversal.

Frailty, as a clinical condition, is particularly relevant in hospital settings, where its prevalence is higher. The literature highlights the need for specific clinical pathways for its management in these environments [25,26,27,28], which underscores the relevance of Moving4notfrail^®^ as a structured tool to guide rehabilitation nursing practice. Its multicomponent and progressive nature is consistent with the International Conference on Frailty and Sarcopenia Research (ICFSR) Expert Consensus Guidelines, which recommend strength, balance, and mobility training as key components of interventions for frail older adults [29].

Recent evidence indicates that multicomponent interventions based on physical exercise are effective in preventing and reversing frailty [30,31,32], with benefits extending beyond the physical domain to cognitive, emotional, and even economic spheres [33].

Moving4notfrail^®^ aligns with these recommendations by proposing an intervention plan focused on muscle strength and gait training, which are key components of the frailty phenotype described by Fried et al. [11]. Its division into five steps facilitates individualised progression, in line with the principles of safe, person-centred rehabilitation. The selection of functionally relevant exercises—incorporating multi-joint, resisted movements that mirror patterns of activities of daily living—supports the restoration of autonomy in self-care [34,35,36].

However, a recent study highlights the gap in access to appropriate rehabilitation care for frail older adults—a challenge that Moving4notfrail^®^ seeks to address by offering a progressive, structured approach that is sensitive to the individual characteristics and preferences of hospitalised older adults [37].

In this sense, Moving4notfrail^®^ not only aligns with international guidelines but also reinforces the specific role of Specialist Nurses in Rehabilitation Nursing in designing, implementing, and evaluating structured, evidence-based exercise programmes within hospital contexts.

When compared to existing programmes such as VIVIFRAIL [38], which focus mainly on community-dwelling and institutionalised older adults, Moving4notfrail^®^ distinguishes itself by being specifically developed for hospitalised frail patients. This hospital-focused and person-centred design represents an innovation within Rehabilitation Nursing practice.

The programme’s structure follows the guidelines of the National Strength and Conditioning Association and the American College of Sports Medicine [39,40], ensuring its scientific foundation and safety in implementation. One of its most innovative aspects is the integration of cognitive and motivational stimulation strategies, including dual-task activities, sensory cues, and verbal and tactile prompts—resources that foster active engagement and enhance overall functionality [41,42,43,44].

Research indicates that exercise programmes for frail or pre-frail older adults—whether hospitalised, institutionalised, or living in the community—do not always provide precise details about their prescription methods. However, these programmes may range from five consecutive days to six months in duration, with a frequency of two to five sessions per week, and although the prescribed intensity varies, a gradual increase is strongly recommended [30].

In addition, incorporating individual preferences contributes to greater motivation and adherence, which are essential elements for the effectiveness and sustainability of the intervention [33]. The final programme prototype, which includes photographic illustrations of all exercises, enhances its potential for practical application by health professionals and supports greater engagement of older adults and caregivers.

This aspect also supports its future transfer to other contexts—such as long-term care units, day centres, or even home settings—facilitating continuity of care and helping to prevent functional decline after hospital discharge [42,45]. This transition is crucial, given that the effects of hospital-associated frailty may persist, negatively impacting the individual, the caregiver, and the community [46].

In addition to validating the content of Moving4notfrail^®^, this study makes a pioneering contribution to the systematisation of the role of the SNRN in addressing frailty in hospital settings. Although there are guiding documents from the Portuguese Order of Nurses recognising the specificity of this role [14,20], there has, until now, been no structured, validated, and operational tool. Moving4notfrail^®^ emerges as a concrete response to this gap, offering a practical, evidence-based instrument validated by experts. Its implementation contributes to translating theoretical nursing knowledge into measurable clinical outcomes, strengthening professional autonomy and enhancing the visibility of Rehabilitation Nursing in multidisciplinary care contexts. Furthermore, Moving4notfrail^®^ has the potential to raise awareness among nursing professionals about the need to rethink current hospital practices to improve the quality of care provided [47].

Finally, this proposal aligns with the recommendations of a recent study highlighting the importance of having clear and implementable guidelines for the management of frailty in hospital settings. Such guidelines can help overcome barriers such as limited time for planning and delivering care, insufficient knowledge among health professionals on the topic, and the perception that substantial resources are required to intervene in this clinical condition [27].

### 4.1. Implications for Practice

Moving4notfrail^®^ has the potential to transform the approach to hospitalised older adults with physical frailty. Its systematic implementation could lead to a significant reduction in complications associated with immobility, such as physical decline, falls, loss of autonomy, and prolonged hospital stays. Moreover, it provides SNRN with a validated tool that reinforces and enhances their role, supporting safer, more effective, and person-centred practice.

As an accessible and adaptable programme, it also has the potential to contribute to a more comprehensive response to the needs of older adults.

### 4.2. Limitations and Future Recommendations

Despite the methodological rigour adopted in all phases of the study, some limitations should be acknowledged. The intentional sampling of experts may limit the diversity of perspectives and restrict the generalisability of the results. Furthermore, this study focused on the development and content validation of Moving4notfrail^®^, highlighting the need for future research to assess its applicability in real-world settings, its clinical outcomes (e.g., improvements in muscle strength, gait speed, and independence in self-care activities), and its impact on the quality of life of older adults.

It is recommended that the programme be implemented in hospital units, followed by evaluations of its effectiveness through quasi-experimental or experimental designs with pre- and post-intervention measures. Analysing the acceptability of the programme by professionals, older adults, and caregivers will also be essential to support its sustainable and large-scale implementation.

We also acknowledge that the future integration of the programme into real clinical settings may benefit from interprofessional collaboration, particularly with physiotherapists or clinical exercise specialists, especially in units that already have multidisciplinary rehabilitation pathways or protocols in place. This stage, which goes beyond the scope of the present study, may constitute a future investment, further strengthening the programme’s implementation potential across different settings and within a multidisciplinary team.

## 5. Conclusions

This study enabled the rigorous methodological development and validation of Moving4notfrail^®^, a rehabilitation nursing programme designed for hospitalised older adults with physical frailty. Structured in five progressive steps, with exercises adapted to the functional capacity of each individual, the programme offers a concrete response to the needs identified in this population and to the demands of specialised rehabilitation nursing practice.

The content validation of Moving4notfrail^®^ by experts, through a modified e-Delphi, demonstrated a high level of agreement regarding the relevance, applicability, and clarity of the programme components. This evidence highlights its potential to prevent the worsening of frailty during hospitalisation and promote functionality and autonomy in older adults. The inclusion of motivational and cognitive strategies, such as dual-task activities and sensory, visual, verbal, and tactile stimuli, further reinforces the innovative character of Moving4notfrail^®^.

This programme represents a valuable contribution to the systematisation of the intervention of the SNRN, offering a grounded, validated, and user-friendly tool. Beyond the hospital setting, its adaptable format allows for transfer to other care contexts, including home-based care, promoting continuity of care and healthy ageing. Its sustained implementation could represent a significant step towards enhancing the quality of care provided to older adults experiencing physical frailty.

## Figures and Tables

**Table 1:** Sociodemographic and professional profile of the experts.

Sociodemographic and Professional Characteristics	First and Second Rounds (n = 18)
Gender n (%)	
Female	11 (61.1%)
Male	7 (38.9%)
Age (years) Mean; Std. Dev.	38.7; ±7.7
Education n (%)	
Bachelor’s degree	7 (38.9%)
Master’s degree	11 (61.1%)
Job Title n (%)	
Nurse	5 (27.8%)
Nurse Specialist	13 (72.2%)
Area of specialization in Nursing n (%)	
Rehabilitation nursing	18 (100%)
Time of professional practice (years) Mean; Std. Dev.	15.8; ±7.8
Time of professional practice as a rehabilitation nursing specialist (years) Mean; Std. Dev.	7.9; ±3.6

**Table 2:** Content validation of the components of the “Moving for not frail: rehabilitation nursing programme”.

Programme Components	CVI First Round	CVI Second Round
Programme steps	1.00	1.00
Resources used in the programme	0.89	1.00
Session frequency	1.00	1.00
Session duration	1.00	1.00
Variety of exercises	0.89	1.00
Exercise progression	1.00	1.00

CVI—Content Validity Index.

## Data Availability

The data that support the findings of this study are available from the corresponding author upon reasonable request.

## Public Involvement Statement

No public involvement in any aspect of this research.

## Guidelines and Standards Statement

This manuscript was drafted against the CREDES (Conducting and Reporting Delphi Studies) guidance for Delphi study research.

## Abbreviations

The following abbreviations are used in this manuscript:

SNRN	Specialist Nurses in Rehabilitation Nursing
WHO	World Health Organization
CVI	Content Validity Index
